# Using a peanut ball during labour versus not using a peanut ball during labour for women using an epidural: study protocol for a randomised controlled pilot study

**DOI:** 10.1186/s40814-018-0346-9

**Published:** 2018-10-04

**Authors:** Virginia Stulz, David Campbell, Biing Yin, Wafa Al Omari, Robin Burr, Heather Reilly, Kenny Lawson

**Affiliations:** 1 School of Nursing and Midwifery Centre for Nursing and Midwifery Research, Nepean Hospital, 1st level Court Building, Derby St, Kingswood, NSW 2340 Australia; 2 Nepean Hospital, Derby St, Kingswood, NSW 2340 Australia; 3 Lithgow Hospital, Corner Col Drewe Drive and Great Western Highway, Lithgow, NSW 2790 Australia; 4 Centre for Nursing and Midwifery Research, Nepean Hospital, 1st level Court Building, Derby St, Kingswood, NSW 2340 Australia; 5 Translational Health Research Institute, Building 3, David Pilgrim Avenue, Campbelltown, NSW 2560 Australia

**Keywords:** Peanut ball, Labour, Epidural

## Abstract

**Background:**

The peanut ball has only been recently used as a support for women labouring with epidurals. The peanut ball is shaped like a peanut and fits snugly between the woman’s legs so that both legs are maintained as opening the pelvic outlet to increase the progress of labour and facilitate descent of the fetal head. Using position changes during labour to enhance widening of the pelvic outlet can be beneficial but a woman who has an epidural is limited in the number of positions she can adopt. No randomised controlled trial has been implemented in Australia to establish the effectiveness of a peanut ball specifically for women using epidurals during labour, and this project addresses this gap. The main aim of this pilot study is to assess the feasibility and practicality of conducting and replicating this trial to a definitive randomised controlled trial (RCT).

**Methods:**

A minimum number of 50 women (25 in each trial arm), who are using an epidural in labour at two hospitals in NSW over a 1-year period, will be recruited and randomly allocated into a group that uses the peanut ball or into a group that does not use the peanut ball. Primary study objectives include assessing the proportion of women willing to be randomised, retention/attrition rates, and with associated reasons. Data will be collected on key clinical outcomes (natural birth rate, length of stay) with means and variances estimated between trial arms. This will inform the appropriate powering of a future definitive RCT. Secondary study objectives will include investigating the completion and acceptability of health and satisfaction surveys and assess the feasibility of conducting an economic evaluation alongside a future trial.

**Discussion:**

This is a two-armed randomised controlled pilot trial. Outcomes from this pilot will inform a larger trial at a tertiary hospital.

**Trial registration:**

Australian New Zealand Clinical Trials Registry, ACTRN12618000662268

**Electronic supplementary material:**

The online version of this article (10.1186/s40814-018-0346-9) contains supplementary material, which is available to authorized users.

## Background

Epidurals have been associated with higher interventions during labour, including a higher incidence of instrumental births [1–3] especially in women having a baby for the first time [4]. Vacuum births have more than doubled in women experiencing epidurals [1]. The National Institute for Health and Care Excellence Guidelines [5] states that there is no evidence pertaining to using a birth ball (the peanut ball falls into the category of a birth ball) during labour, and this project addresses this gap. This guideline [5] recommends that women should be encouraged to move and adopt whatever position she finds comfortable in labour. In fact, there is no evidence in Australia for using a peanut ball specifically for women using epidurals during labour.

The peanut ball is thought to enhance the progress of labour by optimally positioning the fetus in relation to the pelvis [6]. There are multiple benefits associated with maternal position changes, including increased maternal-fetal circulation, decreased pain, improved quality of uterine contractions, facilitation of fetal descent and decreased length of labour [7]. Apart from the physiological benefits of birth, other benefits include less risk of postpartum haemorrhage [8], improved maternal–infant bonding [9], less psychological morbidity postnatally [10], increased rates of successful breastfeeding [9, 11] and improved maternal satisfaction [12]. The woman is also able to independently care for her baby following the birth, whereas women having a caesarean may require more assistance with feeding and general care of the baby. Therefore, widening the pelvic outlet is one way of supporting natural progression of birth.

Three randomised control trials have been implemented in the USA on the use of a peanut ball during labour [13–15]. Of the three randomised control trials in the USA, one of the randomised control trials included women who were scheduled for elective induction of labour and also who used an epidural for labour pain. It was found that the length of time in the first stage of labour was significantly shorter for primiparous (first time having a baby) women using the peanut ball when compared with multiparous (having already had one baby) women and the peanut ball did not make any difference for either group in the time spent pushing [14].

The other randomised control trial showed that women who used the peanut ball during labour had clinically significant lower caesarean section rates, lower instrumental births including forceps and vacuum births, and lower third and fourth degree perineal laceration rates. Even though the findings were clinically significant, they were not statistically significant. There was no difference in the length of stages of labour [15]. One of the randomised control trials showed that using the peanut ball was associated with a significantly lower incidence of caesarean surgery (OR = 0.41, *p* = .04) and is potentially a successful intervention to help progress labour and support vaginal birth for women labouring with an epidural anaesthesia. A small group of nonrandomized labouring women with an epidural who used a peanut ball (*n* = 30) were compared to those who did not (*n* = 22). Lengths of first and second stages of labour were recorded. Results demonstrated a 46-min reduction in first-stage labour and an 11-min reduction in second-stage labour with women who used the peanut ball [13].

The main aim of this pilot study is to assess the feasibility and practicality of conducting and replicating this trial to a definitive randomised controlled trial (RCT) in terms of the rate of willingness to be randomised, retention or attrition rate, staying in the allocated group and reasons for ceasing to use the peanut ball. Data will also be collected on the likely primary and secondary outcome measures to ensure appropriate powering of the future definitive RCT and the minimum clinically important differences between the control and intervention groups. The secondary objectives will investigate completion and acceptability of the health and satisfaction surveys by women who use the peanut ball and all women about their general level of health. Descriptive statistics will be analysed to show key clinical outcomes and the Mann-Whitney *U* and chi-square analyses for differences between the control and intervention groups that will demonstrate an effect size to calculate the appropriate sample size for the definitive RCT. Analyses will be performed blind to group allocation. The study will also include economic measures for costs and health-related quality of life to assess the feasibility of conducting an economic evaluation in a future definitive trial.

There is a need to further investigate these outcomes in Australia as Australia’s practising midwives are a distinct profession compared with obstetric nurses in the USA, so the US results cannot be generalised to Australia. The numerous midwifery-led models of care in Australia [16] (as compared with those of the USA) means that midwives embrace autonomy in their work and promote non-pharmacological or alternative therapies that would impact on their practice if they suggest the peanut ball when working with women with epidurals in labour. Midwifery-led models of care have identified benefits for women and babies with no adverse effects in collaboration with obstetricians and other health professionals [16].

This study will provide evidence about the effect of using the peanut ball for women who have an epidural during labour and be the first of its kind in Australia. It is envisaged that the results of this study will provide evidence for a larger randomised controlled trial in other hospitals in the Nepean and Blue Mountains Local Health District.

## Methods/design

This pilot study will be implemented at the Blue Mountains Anzac District Memorial and Lithgow hospitals, on a group of low-risk women, over a 1-year time period to assess whether there is sufficient evidence for justification for using the peanut ball in a larger randomised controlled study.

Ethics approval for this study was granted in April 2018 by the Nepean Blue Mountains Local Health District Human Research Ethics committee (HREC/18/Nepean/30) and reciprocal approval from Western Sydney University – REDI Reference: RH12693. The trial was registered with the Australian New Zealand Clinical Trials Registry, ACTRN12618000662268, (http://www.ANZCTR.org.au/ACTRN12618000662268.aspx) on the 24th of April at 11:38 am. The study protocol (version 3, March 21, 2018) (see Additional file 1 SPIRIT Protocol) has been designed in accordance with the SPIRIT guidelines (see Additional file 2). Any change to the trial protocol will be communicated to all investigators, reflected in changes to the trial registry and first approved via the ethics committee.

### Setting, recruitment and informed consent

The trial intervention will be conducted in Sydney, Australia, at the Centre for Nursing and Midwifery Research, Nepean hospital, Western Sydney University. The major sites for this pilot study are the Blue Mountains Anzac District Memorial and Lithgow hospitals. Pregnant primiparous (having first baby) and multiparous (having subsequent baby) women who are labouring with an epidural at the Blue Mountains Anzac District Memorial and Lithgow hospitals will be included in the study.

The participants will be identified and approached and consented during labour if they have an epidural.

The women who fit the inclusion criteria will be provided a participant information sheet and then asked to sign a participant consent by the midwife not involved in her care. Once the woman has consented to the project, she has the opportunity to withdraw from the project at any time throughout the course of the project, and not be penalised. The consent will be placed in a research box in the birthing suite by the midwife so that the researcher will be able to recontact the woman to complete an online survey if she used the peanut ball.

### Eligibility criteria

Women will be eligible to participate in the trial if they present the following:

- At least 36 weeks of gestation with a live fetus
- Cephalic (head–down presentation)

Women will be ineligible to participate if they develop moderate to severe pre-eclampsia, or experience severe essential hypertension, and if they are being treated for insulin-dependent diabetes (gestational or pre-pregnant). Women will also be excluded if the fetal heart rate trace is abnormal or suspicious or if they experience an intra-uterine death.

### Randomisation and allocation concealment

Practitioners and participants will know the allocation group for the women, as the woman will either use the peanut ball or not use the peanut ball, so they will not be blinded. A computerised, internet-based central randomisation service (sealedenvelope.com) will be used to provide randomisation and allocation concealment. The research assistant and other investigators performing data collection, entry and analysis will be blind to group allocation. The women will be randomly assigned to either the control or intervention group by the random allocation selection process that will involve sealed opaque envelopes that only the midwife opens once the woman consents to be involved in the pilot study. If the woman is assigned to the intervention group, she would participate in using the peanut birthing ball, and if she is assigned to the control group, she would not use the peanut birthing ball.

### Treatment schedule

**Fig. 1:**
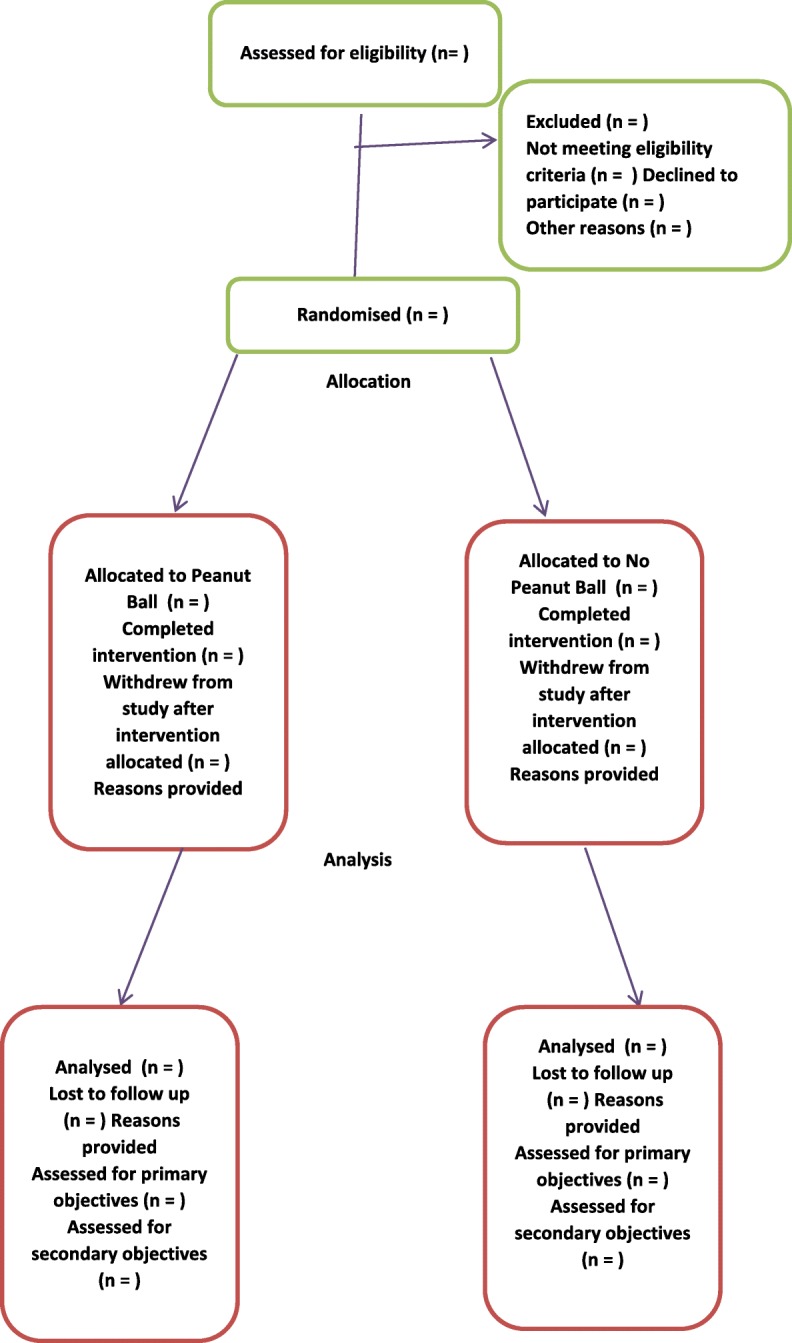
Participant flow peanut ball versus no peanut ball pilot randomised controlled trial.

This study uses the same model (see Fig. 1) as the previously aforementioned three randomised control trials [13–15] in the USA, although Evans and Cremering (2016) only included women having their first babies and Roth et al. (2016) only included women having an elective induction (not spontaneously labouring).

#### Peanut ball

Women in this group will use the peanut ball during labour. The peanut ball is latex free and burst resistant. Ideally, the woman should commence using the peanut ball following insertion of the epidural, when the epidural has taken effect, so that the woman is comfortable and pain-free. It is important to change the woman’s position if she is using the peanut ball during labour with an epidural every 30 min. There are four main positions to be used with the woman having an epidural when she is using the peanut ball.

- *The side lying position* is when the woman is lying on her side, and the peanut ball is wedged between her legs. The top leg lays on the top of the peanut ball curve, and the bottom leg is bent underneath the peanut ball curve. The head of the bed is elevated as much as possible to ensure that the woman is comfortable—see image.
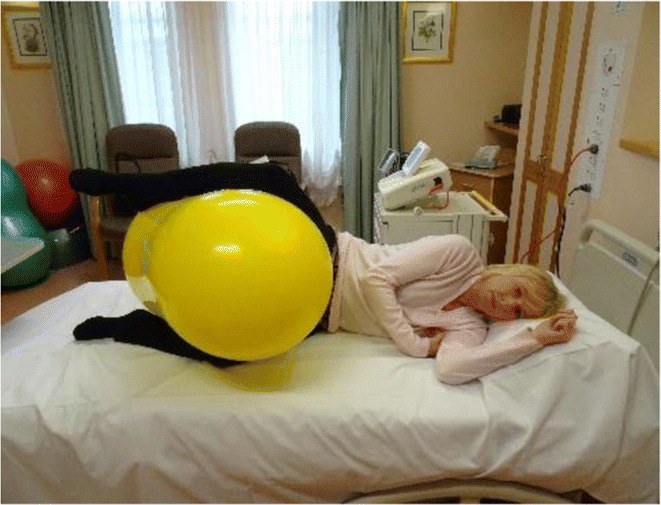
- *The tuck position* is also a side lying position, and the legs are pulled up towards the woman’s head and the ball is brought forward towards the woman’s chest so that the woman can hug the ball with her arms. The head of the bed should also be elevated as much as possible to ensure that the woman is comfortable. This position can also be used for pushing—see image.
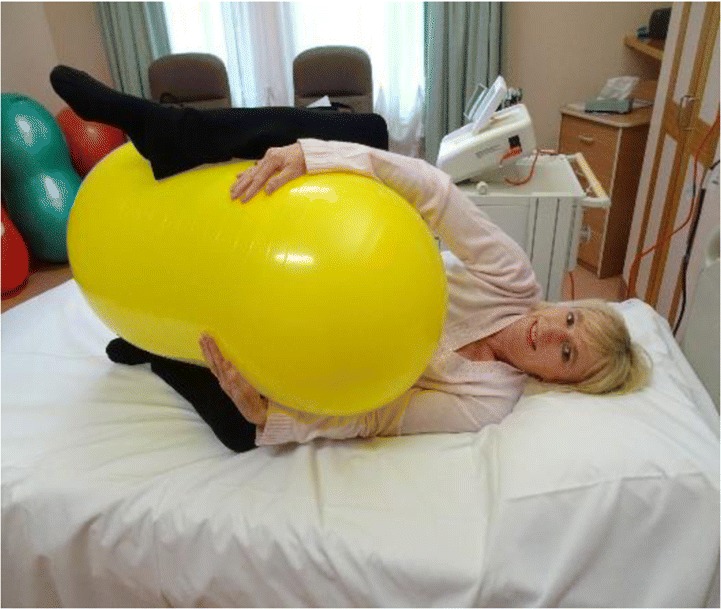
- *The semi sitting position* is when the woman is sitting semi recumbent, and the top leg rests over the peanut ball over the natural curve and the bottom leg is bent and rests under the ball—see image.
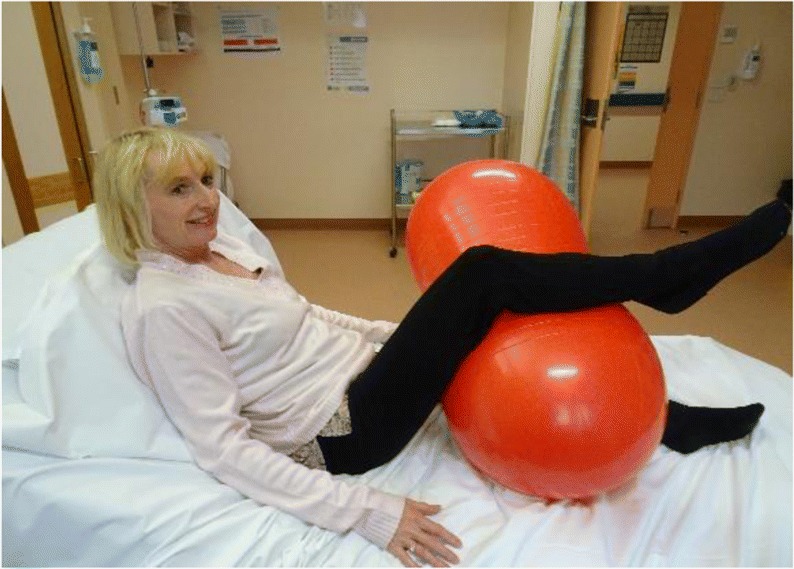
- *The Taylor position* is similar to the semi sitting position, although the legs squeeze the ball and the bottom leg moves up a bit higher towards the woman’s head—see image.
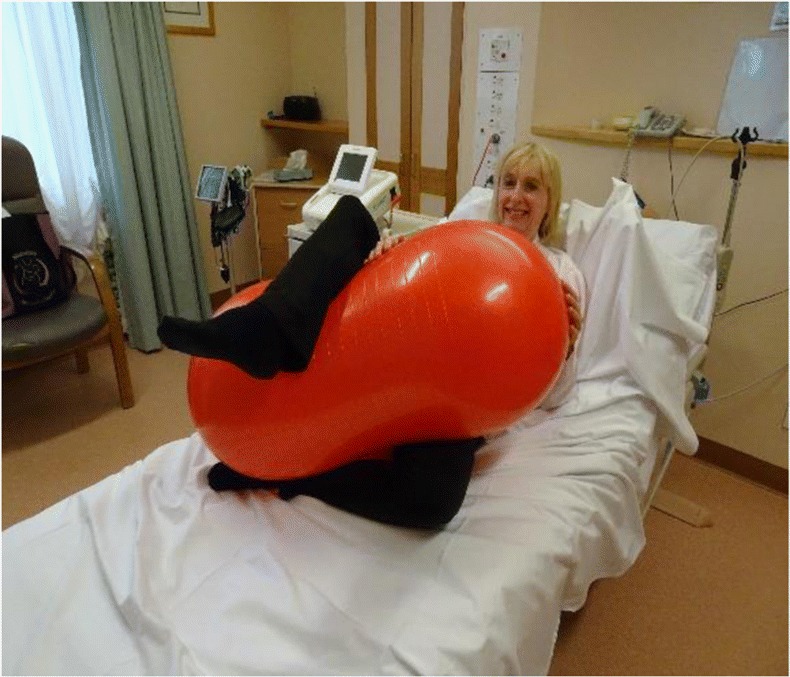

#### Not using peanut ball

The group of women not using the peanut ball will be provided the usual care during labour if using an epidural for pain relief.

### Objectives

The study will have a number of pre-specified measures to address the following objectives, and these measures are detailed for each objective. The primary objectives (see Table 1) relate to progressing the trial to a definitive RCT trial. The secondary objectives (see Table 1) will detail the completion of the survey tools and collection of other variables that are key clinical outcomes when assessing the differences [17] between women using the peanut ball and the group who does not.

**Table 1:** Participant timeline of assessments and interventions

	Study period									
	Enrolment	Allocation	Primip/multip	Other pain relief	Aug/induce	Apgars	Perineum	Position of woman	Cervical dilation at time of epidural insertion	Complete surveys
										Length of stay
Timepoint	During labour	During labour								Postnatal
Enrolment										
Eligibility screen Informed consent	X									
Allocation	X	X								
Interventions										
Intervention		X or								
Comparator group		X								
Assessments										
Primary objectives		X								
Secondary objectives		X	X	X	X	X	X	X	X	X

#### Primary objectives

The primary objectives of the trial include the following:

1. To determine how many women are willing to be randomised into either the control or intervention group
2. To estimate recruitment and attrition rate of women into the study
3. To estimate the number of women staying in the allocated group
4. To determine why women stopped using the peanut ball
5. To assess likely primary and secondary outcome measures to inform sampling size and powering of the definitive RCT

#### Secondary objectives

The secondary objectives include the following:

1. To investigate completion of the health and satisfaction surveys
2. To assess the feasibility of conducting an economic evaluation alongside a future definitive trial
3. To measure key clinical outcomes and differences between the control and intervention group (see Table 1)

Table 1 outlines the participant timeline of assessments and interventions.

### Measures used to achieve objectives

#### Primary objectives

1. *Willingness of women to be randomised into the control or intervention group*
  The measure used to assess this objective will be assessed by the midwife in the birthing unit, and once the woman has an epidural in situ, the woman will then be consented by the midwife. The midwife will then access the sealed opaque envelopes that allocate the woman to the group using the peanut ball or the group receiving standard care and not using the peanut ball (this has been generated by the computer software: sealedenvelope.com). The midwife will have no previous knowledge of which group the woman will be allocated to until the envelope is opened (allocation concealment). If the woman has been allocated to the group not using the peanut ball and she decides she wants to use the peanut ball, the woman’s reasons for using the peanut ball will be noted and the midwife will notate this in the Peanut Ball Research Notebook that accompanies the boxes for information and consent and the allocation box in the birthing suite. The Coordinating Investigator will liaise and visit contacts at both sites for this information.
2. *Estimation of recruitment and attrition rate*
  Acceptability of the recruitment methods will be assessed including any adverse events recorded whilst using the peanut ball during labour (these details will be accessed via the Electronic Medical Record or woman’s progress notes during labour by the coordinating investigator). The main method of recruitment of the women will be midwives working in the birthing unit, identifying and approaching the woman, as they have been previously educated about the research project by education sessions and an education package has also been written for the midwives about using the peanut ball for the research project provided by the coordinating investigator. Sample size analysis should be adequate to estimate the recruitment rate [18]. Modifications may be made to the protocol for the full trial based on recruitment rates.
3. *Estimation of women staying in allocated group*
  Midwives will record in the Peanut Ball Research Notebook in the birthing unit if the woman changes her mind if she is allocated to the control or intervention group and decides to change to the other group other than the group she has been allocated. It is important to achieve an “intention to treat analysis” and that participants are analysed according to the group they are originally allocated [19].
4. *To determine why women stopped using the peanut ball*
  The Coordinating Investigator will access the Electronic Medical Record to assess why the woman stopped using the peanut ball. It is important to achieve an “intention to treat analysis” to determine whether or not they adhered to or accepted the intervention. The importance of reporting of numbers and the reasons why participants were lost to follow up is necessary to assess the extent to what the intention to treat may lead to and provide an underestimate of the efficacy of the intervention under ideal circumstances [19].
5. *To assess likely primary and secondary outcome measures to inform sampling size and powering of the definitive RCT*
  The main purpose of this pilot study is to assess the feasibility of conducting a full definitive trial and not to generate statistically significant results. Nonetheless, a secondary aim is to inform the sample size calculation to design a full definitive trial. This requires an estimate of a clinically meaningful effect size and variance that can be entered into a sample size calculation. The key primary clinical outcome is the rate of vaginal births, and the key secondary outcome is the length of labour. For this pilot study, a decision was taken to opt for 50 pregnant women (25 in each trial arm) to inform this process. Indeed, a previous pilot study that investigated the use of the peanut ball with similar aims determined that this sample size may actually be sufficient to demonstrate statistically significant findings [13]. Therefore, given the high feasibility of recruiting these numbers of women at the study sites, a decision was then made to aim for 50 pregnant women in total (as a minimum), and so opt for a comprehensive pilot trial to ensure the pilot can confidently test feasibility and also inform the powering of a future definitive trial.

#### Secondary objectives

1. *To investigate completion of the health and satisfaction surveys*
  **Health and satisfaction surveys**
  - The Health Questionnaire (EQ-5D) will provide important general physical and mental health information about mobility, self-care, usual activities, (for example, work, study, housework, family or leisure activities), pain, discomfort, anxiety, and depression. EQ-5D is a standardised measure of health status developed by the EuroQol Group in order to provide a simple, generic measure of health for clinical and economic appraisal. The EuroQol Group is a network of international multidisciplinary researchers devoted to the measurement of health status [20]. The Health Questionnaire will also request a single score about level of health experienced on that particular day. The first five questions are measured on a Likert scale, and the last question about perception of health asks the respondent to answer on a scale from 0 to 100 with 0 meaning the worst health they can imagine and 100 meaning the best health they can imagine. It is cognitively undemanding, taking only a few minutes to complete. The survey is applicable to a wide range of health conditions and treatments; it provides a simple descriptive profile and a single index value for health status that can be used in the clinical and economic evaluation of health care as well as in population health surveys. The EQ-5D is designed for self-completion by respondents and is ideally suited for use in postal surveys, in clinics and in face-to-face interviews [20].
  - The survey assessing satisfaction levels obtains information in 13 questions about using the peanut ball in Likert scale responses, yes/no answers and free text responses and takes approximately 5 min to complete. The survey will provide a snapshot about the woman’s experience and enquire about the benefits of using the peanut ball, subsequent use of the peanut ball, whether the woman would recommend using the peanut ball to other women and reasons why, discomfort, specific positions used with the peanut ball, experiencing feelings of empowerment and effect on length of labour and demographic details. The survey was developed in alignment with the results of the previous randomised controlled trials [13–15] and as this research only focuses on quantitative data, it was thought that it was beneficial to obtain some information from women about the comfort, perception and satisfaction about using the peanut ball as complementary evidence to the pilot randomised controlled trial. Face validity has been verified by this survey being reviewed and approved by an ethics committee of expert health research professionals in a local health district in NSW, and the survey will be further validated in this pilot study by comparing responses from both sites by using construct validity and establishing appropriate sample size.
  Both surveys will be distributed to the respondents from an electronic platform—Qualtrics [21] platform—and have been electronically tested to assess the functionality prior to distribution of the online survey. Each survey will be distributed to the participant’s email and de-identified in the computer database. All surveys will be analysed, including those not completed. All of the data collected in this closed survey will be stored in a password-protected computer and will only be accessible by the researchers.
2. *To assess the feasibility of conducting an economic evaluation alongside a future definitive trial*
  The feasibility of collecting data required for an economic evaluation will be tested. For this pilot, we will collect information on health service usage between trial arms, including staff time, medications, procedures and length of stay in hospital. The inclusion of the EQ5D5L will assess the feasibility of incorporating an economic measure of HRQoL, where responses will be converted into ‘health utilities’.
3. *To measure key clinical outcomes and differences between the control and intervention group*
  Details about other birth outcomes will also be collected to determine the baby’s condition, perineal damage, other methods of pain relief used, position of mother and baby during labour, blood loss, cervical dilatation at time of insertion of epidural and evidence of augmentation and/or induction of labour.

### Statistical analysis

#### Quantitative data

In terms of the women’s willingness of being randomised into the control or intervention group and assessment of recruitment and attrition rates and staying in the allocated groups, the Coordinating Investigator will access this information from the Peanut Ball Research Notebook and assess the sample size. This information will be estimated as accurately as possible from the critical parameters we wish to estimate [19, 22] that includes the minimum important differences when comparing the means of continuous outcomes and categorical outcomes between the intervention and control groups. The differences between the vaginal birth rate (assessment of likely primary outcome for definitive RCT) and the length of labour (assessment of likely secondary outcome for definitive RCT) will be analysed. Demographics will be reported using descriptive statistics. The Coordinating Investigator will also access women lost to follow up and if women stopped using the peanut ball from the Electronic Medical Record.

The purpose of the pilot is to assess the feasibility of the component elements together to inform a definitive trial. The pilot and sample size of 50 is not intended to be powered to conduct meaningful statistical tests [19]. Nonetheless, and for exposition purposes, standard tests will be conducted and reported. Online survey results will provide complementary data from the woman’s perspective of using the peanut ball during labour and health and quality of life.

#### Qualitative data

The text responses from the online survey about the woman’s satisfaction about using the peanut ball will be analysed by thematic analysis. Thematic analysis is an iterative and inductive process which will involve researchers reading and identifying and labeling codes in the data, and developing themes and subthemes.

## Discussion

The peanut ball is a non-pharmacological method of pain relief that may be used by women using epidurals in labour, and preliminary randomised controlled trials in the USA have shown clinically and statistically significant findings. Currently, peanut balls are being used by women during labour, in hospitals in Australia; however, there is no existing evidence that the peanut ball makes a difference for women, either as a birthing ball or whilst using an epidural. It is important to fully investigate the potential benefits of using the peanut ball in Australia, especially when we are now working with increased rates of caesarean sections [23].

The web-based survey about the woman’s general health will provide general physical and mental health information about mobility, self-care, usual activities, pain, discomfort, anxiety and depression. We would expect that this cohort of pregnant women with low-risk pregnancies will provide a result of a generally healthy population. The web-based survey about using the peanut ball will provide a snapshot of information about the woman’s level of satisfaction, positions she used, her perception about influencing the length of labour, level of empowerment and whether she would recommend the peanut ball to other women using epidurals during labour. Demographics will also be collected. Expected results in the overall context of the study would show that the peanut ball has benefits for women using epidurals in labour, not only in her positive perception and feeling of empowerment but also increasing her comfort.

This study will be the first to investigate the pilot effect of the peanut ball for women using epidurals in labour in a low-risk maternity model of care in Australia. It is expected that the mean clinical and health service outcomes will improve in the peanut ball arm. If the results point to reject using the peanut ball and no benefits are found, it is expected that midwives will continue to suggest using the peanut ball to increase comfort and empowerment for women using epidurals in labour. The pilot trial will inform the development of a fully powered definitive trial to assess the clinical meaningful and statistically significant outcomes, including an economic evaluation to assess cost effectiveness.

### Trial status

Recruitment has not yet commenced.

## Additional files

Additional file 1: SPIRIT Checklist. (DOCX 59 kb)

Additional file 2: SPIRIT Protocol. (PDF 122 kb)

## Abbreviation

RCT: Randomised controlled trial
