# Supplementary material for: Using a peanut ball during labour versus not using a peanut ball during labour for women using an epidural: study protocol for a randomised controlled pilot study
Source: Pilot Feasibility Stud. 2018 Oct 4;4:156. doi: 10.1186/s40814-018-0346-9 (PMC6171141; doi:10.1186/s40814-018-0346-9)
Supplement: Supplementary file 1 — SPIRIT Checklist. (DOCX 59 kb) [file 40814_2018_346_MOESM1_ESM.docx]

**Additional File 1 - SPIRIT Checklist**

**Administrative Information**

**Title Item No 1** Using a peanut ball during labour versus not using a peanut ball during labour for

women using an epidural: study protocol for a randomised controlled pilot study

**Trial registration Item No 2a** Australian New Zealand Clinical Trials Registry, ACTRN12618000662268 (<http://www.ANZCTR.org.au/ACTRN12618000662268.aspx>).

**No 2b** All items completed from the World Health Organization Trial Registration Data Set

**Protocol version Item No 3** Version 3 21^st^ March

**Funding Item No 4** Partnership grant between Western Sydney University and Nepean Blue Mountains Local Health District

**Roles and responsibilities Item No 5a** Associate Professor Virginia Stulz, Western Sydney University

Principle activities include education initially about the use of the peanut ball for midwives in the

birthing suite of Lithgow and Katoomba hospitals and overseeing the entire project. This includes

ensuring appropriate allocation of women to either intervention or control groups, data collection,

confidentiality of data and statistical analysis of final results.

Associate Professor Kenny Lawson, Translational Health Research Institute

Dr Lawson will be responsible for the conduct cost effectiveness analysis, producing preliminary

results from the pilot, making the business case for a larger trial, and undertaking a comprehensive

cost effectiveness analysis from the full trial. He will also lead on publications concerning the

economic and co-author relevant manuscripts.

Dr David Campbell, Nepean and Blue Mountains Anzac Memorial hospital

Assisting with recruitment and final writing of publications.

Dr Biing Yin, Lithgow hospital

Recruitment and education at Lithgow and final writing of publications.

Dr Wafa Al Omari, Nepean and Blue Mountains Anzac Memorial hospital

Assistance with education and recruitment and final writing publications.

Dr Robin Burr, Nepean hospital

Dr Burr's involvement will include continual consultation and expertise from an obstetric perspective whilst the study is in progress.  Dr Burr will be a key author on publications. Dr Burr will be involved

in the future planning of the randomised control study at Nepean hospital to follow.

Justine Elliott, Nepean hospital

Justine will be involved in the education of the midwives in the use of the peanut ball at both the

Blue Mountains and Nepean hospitals.

Madeleine Simpson, Blue Mountains Anzac Memorial hospital

Madeleine will be responsible for providing midwifery expertise and knowledge on site during the

pilot study and assisting with data collection in liaison with the Principal Investigator.

Heather Borradale, Private hospital, Queensland

Heather has presented a poster at a recent national conference detailing use of the peanut ball. She works in a hospital that uses the peanut ball for women labouring with epidurals.

Deborah Gaynor

Deborah will be directly responsible for education of the midwives at Lithgow hospital in the use of

the peanut ball and provide midwifery expertise and knowledge on site during the pilot study.

Heather Reilly

Heather has had substantial research experience performing literature reviews, data collection and analysis of data in her previous research roles supporting professorial positions.

**Item No 5b** N/A

**Item No 5c** N/A

**Item No 5d** N/A

**Introduction**

**Background and rationale Item No 6a**

This pilot study would be implemented at the Blue Mountains Anzac Memorial and Lithgow hospitals, on a group of low risk women, to assess whether there is sufficient evidence for justification for using the peanut ball in a larger randomised controlled study. There is a need to further investigate these outcomes in Australia as Australia’s practising midwives are a distinct profession compared with Obstetric Nurses in the United States, so the United States results cannot be generalized to Australia. The National Institute for Health and Care Excellence Guidelines (NICE, 2017)^1^ states that there is no evidence pertaining to using a birth ball (the peanut ball falls into the category of a birth ball) during labour and this project addresses this gap. In fact, there is no evidence in Australia for using a peanut ball specifically for women using epidurals during labour.

This study will provide evidence about the effect of using the peanut ball for women who have an epidural during labour and be the first of its kind in Australia. It is envisaged that the results of this study will provide evidence for a larger randomised controlled trial in other hospitals in the Nepean and Blue Mountains Local Health District.

The main aim of this pilot study is to assess the feasibility and practicality of conducting and replicating this trial to a definitive randomised controlled trial (RCT) in terms of the rate of willingness to be randomised, retention or attrition rate, staying in the allocated group and reasons for ceasing to use the peanut ball. Data will also be collected on the likely primary and secondary outcome measures to ensure appropriate powering of the future definitive RCT and the minimum clinically important differences between the control and intervention groups. The secondary objectives will investigate completion and acceptability of the health and satisfaction surveys by women who use the peanut ball and all women about their general level of health. Descriptive statistics will be analysed to show key clinical outcomes and the Mann-Whitney U and Chi-square analyses for differences between the control and intervention groups that will demonstrate an effect size to calculate the appropriate sample size for the definitive RCT. Analyses will be performed blind to group allocation. The study will also include economic measures for costs and health related quality of life to assess the feasibility of conducting an economic evaluation in a future definitive trial.

The peanut ball has only been recently used as a support for women labouring with epidurals in situ. Originally, the peanut ball had been used for physical therapy. The peanut ball is shaped like a peanut and fits snugly between the woman’s legs so that both legs are maintained as opening the pelvic outlet to increase the progress of labour and facilitate descent of the fetal head (Grant & Clutter, 2014)^2^.

The peanut ball is shaped like a peanut shell where the middle circumference is smaller than the ends of the ball. In order to mimic the desired upright position during birth, the peanut ball can be used whilst the woman is in bed and the ball is supported by a pillow placed behind the woman’s hips to support her legs. Using position changes during labour to enhance widening of the pelvic outlet can be beneficial (Tussey, Botsios, Gerkin, Kelly, Gamez & Mensik, 2015)^3^ but a woman who has an epidural is limited in the number of positions she can adopt. The peanut ball is thought to enhance the progress of labour by optimally positioning the fetus in relation to the pelvis (Johnston, 1997)^4^. There are multiple benefits associated with maternal position changes, including increased maternal-fetal circulation, decreased pain, improved quality of uterine contractions, facilitation of fetal descent and decreased length of labour (Zwelling, 2010)^5^. Apart from the physiological benefits of birth, other benefits include less risk of postpartum haemorrhage (Davis, Baddock, Pairman et al., 2012),^6^ improved maternal–infant bonding (Moore, Anderson & Bergman 2007),^7^ less psychological morbidity postnatally (Michels, Kruske & Thompson 2013),^8^ increased rates of successful breastfeeding (Moore et al., 2007; Brown & Jordan, 2013) ^9,10^ and improved maternal satisfaction (Leap, Sandall, Buckland & Huber 2010)^11^. The woman is also able to independently care for her baby following the birth, whereas women having a caesarean may require more assistance with feeding and general care of the baby. Therefore widening the pelvic outlet is one way of supporting natural progression of birth.

There is limited evidence on the use and effectiveness of a peanut ball for pregnant women during labour, especially in Australia. In fact, no randomised controlled trial has been implemented in Australia to establish the effectiveness of a peanut ball for labouring women. Three randomised control trials have been implemented in the United States of America on the use of a peanut ball during labour (Tussey et al., 2015; Roth, Dent, Parfitt, Hering & Bay, 2016; Evans & Cremering, 2016)^3,12,13^ and four randomized control trials were conducted in Brazil, Spain, Taiwan and Iran to establish the effect of a birthing ball, but not specifically a peanut ball (Makvandi, Roudsari, Sadeghi & Karimi, 2015) ^14^.

Of the three randomised control trials in the United States of America, one of the randomised control trials included women who were scheduled for elective induction of labour and also who used an epidural for labour pain. It was found that the length of time in first stage of labour was significantly shorter for primiparous (first time women having a baby) women using the peanut ball when compared with multiparous (having already had one baby) women and the peanut ball did not make any difference for either group in the time spent pushing (Roth et al., 2016) ^12^.

The other randomized control trial showed that women who used the peanut ball during labour had clinically significant lower caesarean section rates, lower instrumental births including forceps and vacuum births; and lower third and fourth degree perineal laceration rates. Even though the findings were clinically significant, they were not statistically significant. There was no difference in the length of stages of labour (Evans & Cremering, 2016) ^13^. One of the randomised control trials showed that using the peanut ball was associated with a significantly lower incidence of caesarean surgery (OR = 0.41, p = .04) and is potentially a successful intervention to help progress labour and support vaginal birth for women labouring with an epidural anaesthesia (Tussey et al., 2015) ^3^.

Epidurals have been associated with higher interventions during labour, including a higher incidence of instrumental births (Anim-Somuah Smyth & Jones, 2011; Leighton & Halpern, 2002; Lieberman & O'Donoghue, 2002),^15-17^ especially in women having a baby for the first time (Comparative Obstetric Mobile Epidural Trial Study Group, 2001)^18^. Vacuum births have more than doubled in women experiencing epidurals (Anim-Somuah et al., 2011) ^15^.

Instrumental vaginal births are associated with an increased risk of perineal damage, urinary incontinence, painful sexual intercourse and bowel and sexual problems (Eason, Labrecque, Wells, & Feldman, 2000; Ekeus, Nilsson, & Gottvall, 2008; Groutz, Cohen, Gold, Hasson, Wengier, Lessing & Gordon, 2011) ^19-21^. Instrumental vaginal births are also associated with adverse events in infants, such as cephalhaematoma or caput succedaneum and skull fractures with vacuum births (Simonson, Barlow, Dehennin, Sphel, Toppet, Murillo & Rozenberg, 2007) ^22^.

There are no expected harms in using the peanut ball intervention.

**Item No 6b**

The comparators will not use the peanut ball as we want to compare the outcomes between the group that uses the peanut ball and the group not using the peanut ball.

**Objectives Item no 7**

The study will have a number of pre-specified or measures to address the following objectives and these measures are detailed for each objective. The primary objectives (see Figure for Item No 13) relate to progressing the trial to a definitive RCT trial. The secondary objectives (see Figure for Item No 13) will detail completion of the survey tools and collection of other variables that are key clinical outcomes when assessing the differences (Eldridge, Chan, Campbell, Bond, Hopwell, Thabane & Lancaster, 2016)^23^ between women using the peanut ball and the group who does not.

***Primary objectives***

The primary objectives of the trial include the following:

1. To determine how many women are willing to be randomised into either the control or intervention group
2. To estimate recruitment and attrition rate of women into the study
3. To estimate the number of women staying in the allocated group
4. To determine why women stopped using the peanut ball
5. To assess likely primary and secondary outcome measures to inform sampling size and powering of the definitive RCT

***Secondary objectives***

The secondary objectives include the following:

1. To investigate completion of the health and satisfaction surveys
2. To assess the feasibility of conducting an economic evaluation alongside a future definitive trial
3. To measure key clinical outcomes and differences between the control and intervention group (see Figure for Item No 13)

**Trial design Item no 8**

The allocation to intervention will be a randomised control trial. If the woman is assigned to the intervention group, she would participate in using the peanut birthing ball and if she is assigned to the control group, she would not use the peanut birthing ball. The assignment will include a single group where all participants in the intervention group receive the same intervention.

**Methods: Participants, interventions, and outcomes**

**Study setting Item no 9**

The data will be collected in Australia only at two sites- Blue Mountains Anzac District Memorial hospital and Lithgow hospital.

**Eligibility criteria Item no 10**

The sample will include women who are at least 36 weeks gestation with a live fetus and present with a cephalic presentation. Women will be excluded if they develop moderate to severe pre-eclampsia, or experience severe essential hypertension and if they are being treated for insulin dependent diabetes (gestational or pre-pregnant). Women will also be excluded if the fetal heart rate trace is abnormal or suspicious or if they experience an intra-uterine death.

**Interventions Item no 11a**

**Treatment schedule**

**Peanut ball** Women in this group will use the peanut ball during labour. Ideally the woman should commence using the peanut ball following insertion of the epidural, when the epidural has taken effect, so that the woman is comfortable and pain-free. It is important to change the woman’s position if she is using the peanut ball during labour with an epidural every 30 minutes. There are four main positions to be used with the woman having an epidural when she is using the peanut ball.

- ***The side lying position*** is when the woman is lying on her side and the peanut ball is wedged between her legs. The top leg lays on the top of the peanut ball curve and the bottom leg is bent underneath the peanut ball curve. The head of the bed is elevated as much as possible to ensure that the woman is comfortable.
- ***The tuck position*** is also a side lying position and the legs are pulled up towards the woman’s head and the ball is brought forward towards the woman’s chest so that the woman can hug the ball with her arms. The head of the bed should also be elevated as much as possible to ensure that the woman is comfortable. This position can also be used for pushing.
- ***The semi sitting position*** is when the woman is sitting semi recumbent and the top leg rests over the peanut ball over the natural curve and the bottom leg is bent and rests under the ball.
- ***The Taylor position*** is similar to the semi sitting position, although the legs squeeze the ball and the bottom leg moves up a bit higher towards the woman’s head.

**Not using peanut ball** The group of women not using the peanut ball will be provided the usual care during labour if using an epidural for pain relief.

**Item no 11b** Midwives have been directed to document any reasons why the woman discontinued using the peanut ball. Women will also be excluded if the fetal heart rate trace is abnormal or suspicious.

**Item no 11c** An education package for the use of a peanut ball during labour with an epidural specifically for this research project has been introduced for the midwives and doctors caring for the women using the peanut ball. The Coordinating Investigator will liaise with a site investigator to ensure appropriate allocation to a specific group.

**Item no 11d** No other concomitant care relevant during the trial

**Outcomes**

**Item no 12**

**Measures used to achieve objectives**

***Primary objectives***

1. Willingness of women to be randomised into control or intervention group

The measure used to assess this objective will be assessed by the midwife in the birthing unit, and once the woman has an epidural in situ, the woman will then be consented by the midwife. The midwife will then access the sealed opaque envelopes that allocate the woman to the group using the peanut ball or the group receiving standard care and not using the peanut ball (this has been generated by the computer software: sealedenvelope.com). The midwife will have no previous knowledge of which group the woman will be allocated to until the envelope is opened (allocation concealment). If the woman has been allocated the group not using the peanut ball and she decides she wants to use the peanut ball, the woman’s reasons for using the peanut ball will be noted and the the midwife will notate this in the Peanut Ball Research Notebook that accompanies the boxes for information and consent and the allocation box in the birthing suite. The Coordinating Investigator will liaise and visit contacts at both sites for this information.

1. Estimation of recruitment and attrition rate

Acceptability of the recruitment methods will be assessed including any adverse events recorded whilst using the peanut ball during labour (these details will be accessed via the Electronic Medical Record or woman’s progress notes during labour by the coordinating investigator). The main method of recruitment of the women will be midwives working in the birthing unit, identifying and approaching the woman, as they have been previously educated about the research project by education sessions and an education package has also been written for the midwives about using the peanut ball for the research project provided by the coordinating investigator. Sample size analysis should be adequate to estimate the recruitment rate (Teare, Dimairo, Shephard, Hayman, Whitehead, & Walters, 2014) ^24^. Modifications may be made to the protocol for the full trial based on recruitment rates.

1. Estimation of women staying in allocated group

Midwives will record in the Peanut Ball Research Notebook in the birthing unit if the woman changes her mind if she is allocated to the control or intervention group and decides to change to the other group other than the group she has been allocated. It is important to achieve an “intention to treat analysis” and that participants are analysed according to the group they are originally allocated (Kirkwood & Sterne, 2003)^25^.

1. To determine why women stopped using the peanut ball

The Coordinating Investigator will access the Electronic Medical Record to assess why the woman stopped using the peanut ball. It is important to achieve an “intention to treat analysis” to determine whether or not they adhered to or accepted the intervention. The importance of reporting of numbers and the reasons why participants were lost to follow up is necessary to assess the extent to what the intention to treat may lead to and provide an underestimate of the efficacy of the intervention under ideal circumstances (Kirkwood & Sterne, 2003) ^25^.

1. To assess likely primary and secondary outcome measures to inform sampling size and powering of the definitive RCT

The main purpose of this pilot study is to assess the feasibility of conducting a full definitive trial; and not to generate statistically significant results. Nonetheless, a secondary aim is to inform the sample size calculation to design a full definitive trial. This requires an estimate of a clinically meaningful effect size and variance that can be entered into a sample size calculation. The key primary clinical outcome is the rate of vaginal births and key secondary outcome is the length of labour. For this pilot study a decision was taken to opt for 50 pregnant women (25 in each trial arm) to inform this process. Indeed a previous pilot study that investigated the use of the peanut ball with similar aims determined that this sample size may actually be sufficient to demonstrate statistically significant findings (Tussey et al., 2015)^3^. Therefore, given the high feasibility of recruiting these numbers of women at the study sites, a decision was then made to aim for 50 pregnant women in total (as a minimum); and so opt for a comprehensive pilot trial to ensure the pilot can confidently test feasibility and also inform the powering of a future definitive trial.

***Secondary objectives***

1. To investigate completion of the health and satisfaction surveys

**Health and satisfaction surveys**

1. The Health Questionnaire (EQ-5D) will provide important general physical and mental health information about mobility, self-care, usual activities, (for example, work, study, housework, family or leisure activities), pain, discomfort, anxiety and depression. EQ-5D is a standardised measure of health status developed by the EuroQol Group (1990)^26^ in order to provide a simple, generic measure of health for clinical and economic appraisal. The EuroQol Group is a network of international multidisciplinary researchers devoted to the measurement of health status (The EuroQuol Group, 1990)^26^. The Health Questionnaire will also request a single score about level of health experienced on that particular day. The first five questions are measured on a Likert scale and the last question about perception of health asks the respondent to answer on a scale from 0 to 100 with 0 meaning the worst health they can imagine and 100 meaning the best health they can imagine. It is cognitively undemanding, taking only a few minutes to complete. The survey is applicable to a wide range of health conditions and treatments, it provides a simple descriptive profile and a single index value for health status that can be used in the clinical and economic evaluation of health care as well as in population health surveys. The EQ-5D is designed for self-completion by respondents and is ideally suited for use in postal surveys, in clinics, and in face-to-face interviews (The EuroQuol Group, 1990)^26^.
2. The survey assessing satisfaction levels obtains information in 13 questions about using the peanut ball in Likert scale responses, yes / no answers and free text responses and takes approximately five minutes to complete. The survey will provide a snapshot about the woman’s experience and enquire about benefits of using the peanut ball, subsequent use of the peanut ball, whether the woman would recommend using the peanut ball to other women and reasons why, discomfort, specific positions used with the peanut ball, experiencing feelings of empowerment and effect on length of labour and demographic details. The survey was developed in alignment with the results of the previous randomised controlled trials (Tussey et al., 2015; Roth et al., 2016; Evans & Cremering, 2016) ^3, 12, 13^ and as this research only focuses on quantitative data, it was thought that it was beneficial to obtain some information from women about the comfort, perception and satisfaction about using the peanut ball as complementary evidence to the pilot randomised controlled trial. Face validity has been verified by this survey being reviewed and approved by an ethics committee of expert health research professionals in a local health district in NSW and the survey will be further validated in this pilot study by comparing responses from both sites by using construct validity and establishing appropriate sample size.

Both surveys will be distributed to the respondents from an electronic platform – Qualtrics (2005)^27^ platform and have been electronically tested to assess the functionality prior to distribution of the online survey. Each survey will be distributed to the participant’s email and de-identified in the computer database. All surveys will be analysed, including those not completed.

All of the data collected in this closed survey will be stored in a password protected computer and will only be accessible by the researchers.

1. To assess the feasibility of conducting an economic evaluation alongside a future definitive trial

The feasibility of collecting data required for an economic evaluation will be tested. For this pilot we will collect information on health service usage between trial arms, including: staff time, medications, procedures and length of stay in hospital. The inclusion of the EQ-5D (The EuroQuol Group, 1990)^26^ will assess the feasibility of incorporating an economic measure of HRQoL, where responses will be converted into ‘health utilities’.

1. To measure key clinical outcomes and differences between the control and intervention group

Details about other birth outcomes will also be collected to determine baby’s condition, perineal damage, other methods of pain relief used, position of mother and baby during labour, blood loss, cervical dilatation at time of insertion of epidural and evidence of augmentation and / or induction of labour.

**Participant timeline of assessments and interventions Figure**

**Item no 13**

|  | **STUDY PERIOD** | | | | | | | | | |
| --- | --- | --- | --- | --- | --- | --- | --- | --- | --- | --- |
|  | Enrolment | Allocation | Primip / multip | Other pain relief | Aug/  induce | Apgars | Perineum | Position of woman | Cervical dilation at time of epidural insertion | Complete surveys  Length of stay |
| **TIMEPOINT** | During labour | During labour | | | | | | | | Postnatal |
| **ENROLMENT:**  **Eligibility screen**  **Informed consent**  **Allocation** | X  X | X |  |  |  |  |  |  |  |  |
| **INTERVENTIONS:**  **Intervention**  **Comparator group** |  | X or  X |  |  |  |  |  |  |  |  |
| **ASSESSMENTS**  **Primary objectives**  **Secondary objectives** |  | X    X | X | X | X | X | X | X | X | X |

**Sample size**

**Item no 14**

The sample size calculation to design a full definitive trial requires an estimate of a clinically meaningful effect size and variance. The key primary clinical outcome is the rate of vaginal births and key secondary outcome is the length of labour. For this pilot study a decision was taken to opt for 50 pregnant women (25 in each trial arm) to inform this process. Indeed a previous pilot study that investigated the use of the peanut ball with similar aims determined that this sample size may actually be sufficient to demonstrate statistically significant findings (Tussey et al., 2015)^13^. Therefore, given the high feasibility of recruiting these numbers of women at the study sites, a decision was then made to aim for 50 pregnant women in total (as a minimum); and so opt for a comprehensive pilot trial to ensure the pilot can confidently test feasibility and also inform the powering of a future definitive trial.

**Recruitment**

**Item no 15**

The main method of recruitment of the women will be midwives working in the birthing unit, identifying and approaching the woman, as they have been previously educated about the research project by education sessions and an education package has also been written for the midwives about using the peanut ball for the research project provided by the coordinating investigator. Sample size analysis should be adequate to estimate the recruitment rate (Teare et al., 2014)^24^. Modifications may be made to the protocol for the full trial based on recruitment rates.

**Methods: Assignment of Interventions (for controlled trials)**

**Allocation:**

**Sequence generation Item no 16a**

Random order generation is being done by simple randomisation (created by computer software).

**Allocation concealment mechanism Item no 16b**

Allocation is concealed by sealed opaque envelopes.

**Implementation Item no 16c**

Practitioners and participants will know the allocation group for the women, as the woman will either use the peanut ball or not use the peanut ball, so they will not be blinded. A computerised, internet-based central randomisation service (sealedenvelope.com) will be used to provide randomisation and allocation concealment. The research assistant and other investigators performing data collection, entry and analysis will be blind to group allocation. The women will be randomly assigned to either the control or intervention group by the random allocation selection process that will involve sealed opaque envelopes that only the midwife opens once the woman consents to be involved in the pilot study.

**Blinding (masking) Item no 17a**

Blinded masking will be used for the people analysing the results / data (data analyst).

**Item no 17b**

The midwives recruiting the women will not be blinded as to the allocation to either intervention or control group. The participants will not be blinded as they will know the allocation to using the peanut ball in the intervention group or not using the peanut ball in the control group. Only the researchers analysing the results will be blinded to what group they are analysing.

**Methods: Data collection, management and analysis**

**Data collection methods Item no 18a**

The data for this research project will be collected by accessing the maternity database E-Maternity to record all women’s outcomes for those women in the control and in the intervention groups. The women who use the peanut ball during labour will be asked a short survey in the postnatal period to determine their satisfaction about using the peanut ball. Both groups will also be sent a survey about their general health.

**Item no 18b**

The data for this research project will be collected by accessing the maternity database E-Maternity to record all women’s outcomes for those women in the control and in the intervention groups. The online survey will be sent to the woman via email using the Qualtrics (2005)^27^ software to be completed in the postnatal period. If the woman has not responded within ten days, one reminder email will be sent.

**Data management Item no 19**

All of the data collected will be stored in a password protected computer and will only be accessible by the researchers. This data will be recorded in SPSS (Statistical Package for Social Sciences) – a statistical package for analysis. Qualtrics will only be able to be accessed by the researchers and is password protected. This data will be located in a Centre for Nursing and Midwifery Research. All data will be de-identified and destroyed after a 15 year period.

**Statistical methods Item no 20a**

***Quantitative data***

In terms of the women’s willingness of being randomised into the control or intervention group and assessment of recruitment and attrition rates and staying in the allocated groups, the Coordinating Investigator will access this information from the Peanut Ball Research Notebook and assess the sample size. This information will be estimated as accurately as possible from the critical parameters we wish to estimate (Kirkwood & Sterne, 2003; Qualtrics, 2005)^25,27^ that includes the minimum important differences when comparing the means of continuous outcomes and categorical outcomes between the intervention and control groups. The differences between the vaginal birth rate (assessment of likely primary outcome for definitive RCT) and the length of labour (assessment of likely secondary outcome for definitive RCT) will be analysed. Demographics will be reported using descriptive statistics. The Coordinating Investigator will also access women lost to follow up and if women stopped using the peanut ball from the Electronic Medical Record.

The purpose of the pilot is to assess the feasibility of the component elements together to inform a definitive trial. The pilot and sample size of 50 is not intended to be powered to conduct meaningful statistical tests (Kirkwood & Sterne, 2003)^25^. Nonetheless, and for exposition purposes, standard tests will be conducted and reported. Online survey results will provide complementary data from the woman’s perspective of using the peanut ball during labour and health and quality of life.

**Item no 20b**

***Qualitative data***

The text responses from the online survey about the woman’s satisfaction about using the peanut ball will be analysed by thematic analysis. Thematic analysis is an iterative and inductive process which will involve researchers reading and identifying and labeling codes in the data, and developing themes and subthemes.

**Item no 20c**

The pilot study will follow an intention to treat analysis, whereby all participants’ information in the study will be included.

**Methods: Monitoring**

**Data monitoring Item no 21a**

A data monitoring committee is not needed as the Coordinating Investigator and Research Assistant will be working in collaboration closely to clean the data and monitor the incoming data.

**Item no 21b**

The Coordinating Investigator will have access to the preliminary, interim and final results and make the final decision to terminate the trial based on sufficient sample size and preliminary results to substantiate a larger randomised control trial.

**Harms Item no 22**

If the woman suffers any distress or psychological injury as a result of this research project, she

should contact the research team as soon as possible.   She will be assisted with arranging

appropriate treatment and support.

**Auditing Item no 23**

No extra auditing will be executed due to the small sample size that will be able to be managed by the researchers.

**Ethics and dissemination**

**Research ethics approval Item no 24**

The research protocol has been submitted to the Nepean Blue Mountains Local Health District and has been approved.

**Protocol amendments Item no 25**

All modifications to the protocol will be communicated to all the investigators on the project.

**Consent or assent Item 26a**

The participants will be identified and approached and consented during labour by the midwives at each site if they have an epidural in labour. Midwives have been educated about these processes at each site and they will have research boxes with the available information and consent forms to access for any women having epidurals. They will also have access to separate boxes that will contain the sealed opaque envelopes that will determine the allocation to either control or intervention group. If the woman is allocated to the control group, she will have usual care in labour and if allocated to the intervention group, she will use the peanut ball in labour.

The participants will be identified and approached and consented during labour if they have an epidural. The women who fit the inclusion criteria will be provided a participant information sheet and then asked to sign a participant consent by the midwife not involved in her care.

**Item 26b**

N/A

**Confidentiality Item 27**

All data will be de-identified and destroyed after a 15 year period and will only be shared by the research team until the research project is complete.

**Declaration of interests Item no 28**

N/A

**Access to data Item 29**

The Coordinating Investigator will have access to the final trial dataset, no contractual agreements in place.

**Ancillary and post-trial care Item no 30**

N/A

**Dissemination policy Item no 31a**

Pregnant women who have identified that they would be interested in the research results on their consent form, will be provided an overall report on the research results in layman’s terms.

**Item no 31b**

N/A

**Item no 31c**

N/A

**Informed consent materials Item 32**

The participant will be provided with a participant informed consent form and possibility to withdraw (see attached Participant Information Consent form).

**Biological specimens Item 33**

N/A

**References**

1. The National Institute for Health and Care Excellence Guidelines, (2017), section 835. p. 325, accessed at <https://www.nice.org.uk/guidance/cg190/evidence/full-guideline-pdf-248734770>
2. Grant, C. B. & Clutter, L. B. (2014). The peanut ball: a remarkable labor support tool. *International Doula, 22*(4), pp. 12 – 15.
3. Tussey, C. M., Botsios, E., Gerkin, R. D., Kelly, L. A., Gamez, J. & Mensik, J. (2015). Reducing length of labor and caesarean surgery rate using a peanut ball for women labouring with an epidural, *The Journal of Perinatal Education, 24*(1), pp. 16-24.
4. Johnston J. (1997). Birth balls*. Midwifery Today With International Midwife, (43)*, 59–67.
5. Zwelling, E. (2010). Overcoming the challenges: maternal movement and positioning to facilitate labor progress. *MCN: The American Journal of Maternal/Child Nursing*, *35*(2), 72-78.
6. Davis, D., Baddock, S., Pairman, S., *et al.* (2012). Risk of severe postpartum hemorrhage in low-risk childbearing women in New Zealand: exploring the effect of place of birth and comparing third stage management of labor*, Birth*, *39*, pp. 98-105.
7. Moore, E.R., Anderson, G.C. & Bergman, N. (2007). Early skin-to-skin contact for mothers and their healthy newborn infants (No. 3). Cochrane Database of Systematic Reviews CD003519. [doi:10.1002/14651858](http://dx.doi.org.ezproxy.uws.edu.au/10.1002/14651858).
8. Michels, A., Kruske, S., & Thompson, R. (2013). Women׳s postnatal psychological functioning: the role of satisfaction with intrapartum care and the birth experience *J. Reprod. Infant Psychol.*, *31*, pp. 172-182.
9. Moore, E.R., Anderson, G.C. & Bergman, N. (2007). Early skin-to-skin contact for mothers and their healthy newborn infants (No. 3). Cochrane Database of Systematic Reviews CD003519. [doi:10.1002/14651858](http://dx.doi.org.ezproxy.uws.edu.au/10.1002/14651858).
10. Brown, A. & Jordan, S., (2013). Impact of birth complications on breastfeeding duration: an internet survey, *J. Adv. Nurs*., *69*, pp. 828-839.
11. Leap, N., Sandall, J., Buckland, S., & Huber, U. (2010) Journey to confidence: women׳s experiences of pain in labour and relational continuity of care, *J. Midwifery Women’s Health*, *55*, pp. 234-242.
12. Roth, C., Dent, S. A., Parfitt, S. E., Hering S. L. & Bay, R. C. (2016). Use of the peanut ball during labor, *The American Journal of Maternal Child Nursing, 41*(3), pp. 140-146.
13. Evans, S. J. & Cremering, M. M., (2016). Use of peanut labor ball for pelvic positioning for nulliparous women following epidural anesthesia, *Journal of Obstetric, Gynecologic & Neonatal Nursing*, *45 (3)*, pp. S47.
14. Makvandi, S. Roudsari, R. L., Sadeghi, R. & Karimi, l. (2015). Effect of birth ball on labor pain relief: A systematic review and meta-analysis, *The Journal of Obstetrics and Gynaecology Research,* accessed July, 2017 doi:10.1111/jog.12802
15. Anim-Somuah, M., Smyth, R. M., & Jones, L. (2011). Epidural versus non-epidural or no analgesia in labour. *Cochrane Database of Systematic Reviews,* *(12),* CD000331.
16. Leighton, B. L., & Halpern, S. H. (2002). The effects of epidural analgesia on labor, maternal, and neonatal outcomes: A systematic review. *American Journal of Obstetrics and Gynecology, 186(5, Suppl),* S69-S77. http://dx.doi.org/10.1016/S0002-9378(02)70182-8
17. Lieberman, E., &O'Donoghue, C. (2002). Unintended effects of epidural analgesia during labor: A systematic review. *American Journal of Obstetrics and Gynecology, 186(5, Suppl. Nature),* S31-S68.
18. Comparative Obstetric Mobile Epidural Trial Study Group. (2001). Effect of low-dose mobile versus traditional epidural techniques on mode of delivery: A randomised controlled trial. *Lancet, 358(9275),* 19-23.
19. Eason, E., Labrecque, M., Wells, G., & Feldman, P. (2000). Preventing perineal trauma during childbirth: A systematic review. *Obstetrics and Gynecology,* *95(3),* 464-471.
20. Ekeus, C., Nilsson, E., & Gottvall, K. (2008). Increasing incidence of anal sphincter tears among primiparas in Sweden: A population-based register study. *Acta Obstetrics Gynecology Scandinavia,* *87,* 564-573.
21. Groutz, A., Cohen, A., Gold, R., Hasson, J., Wengier, A., Lessing, J. B., &Gordon, D. (2011). Risk factors for severe perineal injury during childbirth: A case-control study of 60 consecutive cases. Colorectal Disease, *13(8),* e216-219.
22. Simonson C., Barlow P., Dehennin N., Sphel M., Toppet V., Murillo D., Rozenberg S. (2007). Neonatal complications of vacuum-assisted delivery. *Obstetrics and Gynecology, 109*(3), 626–633.
23. Eldridge, S. M., Chan, C. L., Campbell, M. J., Bond, C. M., Hopwell, S., Thabane, L. & Lancaster, G. A. (2016). CONSORT 2010 statement: extension to randomised pilot and feasibility trials. *BMJ,* 355, i5239 <http://dx.doi..org/10.1136/bmj.i5239>
24. Teare, M. D., Dimairo, M., Shephard, N., Hayman, A., Whitehead, A. & Walters, S. J. (2014). Sample size requirements to estimate key design parameters from external pilot randomised controlled trials: a simulation study. *Trials.* *15*(264),1-13 http://www.trialsjournal.com/content/15/1/264
25. Kirkwood, B. R. & Sterne, J. A. C. (2003). *Essential Medical Statistics.* Oxford: Blackwell Science.
26. 20. The EuroQol Group. (1990). EuroQol-a new facility for the measurement of health-related quality of life. *Health Policy,* *16*(3),199-208.

27. Qualtrics. (2005). Copyright Year: 2018, Provo, Utah, USA. [https://www.qualtrics.com](https://www.qualtrics.com/)
